# Using activity triggered e-diaries to reveal the associations between physical activity and affective states in older adult’s daily living

**DOI:** 10.1186/s12966-015-0272-7

**Published:** 2015-09-17

**Authors:** Martina Kanning, Ulrich Ebner-Priemer, Wolfgang Schlicht

**Affiliations:** Department of Sport and Exercise Science, Division I Exercise and Health Science, University of Stuttgart, Nobelstraße 15, 70569 Stuttgart, Germany; Department of Sport and Sport Science and House of Competence, Karlsruhe Institute of Technology, Karlsruhe, Germany; Department of Psychosomatic Medicine and Psychotherapy, Central Institute of Mental Health, University of Heidelberg, Mannheim, Germany

**Keywords:** Ecological momentary assessment, Emotion, Feelings, Within-subject associations, Activities of daily living, Dual mode theory

## Abstract

**Background:**

Evidence suggests that older adults show positive affects after participating in exercise bouts. However, it is less clear, if and how physical activities in daily living enhance affective states, too. This is dissatisfying, as most of older adults’ physical activities are part of their daily living. To answer these questions we used activity-triggered e-diaries to investigate the within-subject effects of physical activity on three dimensions of affective states (valence, energetic arousal, calmness) during everyday life.

**Methods:**

Older adults (*N* = 74) between 50 and 70 years took part in the study during three consecutive days. Physical activity in daily living was objectively assessed using accelerometers. Affects were measured 10 min after a study participant surpassed a predefined threshold for activity or inactivity. The participants were prompted by an acoustic signal to assess their momentary affective states on an e-diary. Data were analyzed with hierarchical multilevel analyses.

**Results:**

Whenever older individuals were more physically active, they felt more energized (energetic arousal) and agitated (calmness). However, they did not feel better (valence). Interestingly, body mass index (BMI) and valence were associated in a significant cross-level interaction. BMI acts as a moderating variable in the way that lower BMI scores were associated with higher levels of valence scores after being physically active.

**Conclusions:**

The innovative ambulatory assessment used here affords an interesting insight to the affective effects of daily activity of older adults. These effects are no simple and no linear ones, i.e. physical activity is not associated with positive affects per se as shown several times in experimental studies with single activity bouts. Rather there is a differentiating association seen as an enhanced feeling of energy and agitation, which is not accompanied by a better feeling. Socio-emotional selectivity theory may support the finding that older individuals are emotionally more stable during their day-to-day life, which might explain the non-significant effect on the affect dimension valence.

## Background

Meta-analyses show consistently that positive affects or emotions are enhanced after older adults are physically active [1–3]. However, most of older adults’ physical activities are activities of daily living, such as walking to the railway station or to the supermarket or gardening [4–6]. In contrast to structured exercise programs or single exercise bouts, most activities of daily living occur automatically and habitually or are performed spontaneously. Although the physical activities of daily living are relevant for the overall volume of physical activity, it is less clear if the older adults experience similar benefits from the physical activity accrued through the activities of daily living.

Physical activities in daily living are most often of low intensity and short duration [5, 7]. Based on the assumptions of the Dual Mode Theory (DMT; [8]), low to moderate intensities are optimal for increasing positive affects. According to the theory, physical activities that require energy delivered by aerobic metabolic processes are accompanied by positive affects, whereas physical activities that require energy delivered by anaerobic metabolic processes result in negative affects. Dual Mode Theory assumes that the intensity of an activity determines the relative influence of two general factors. One factor focuses on cognitions (e.g., self-efficacy, goals, attributions), and the second factor contains interoceptive cues about the physiological conditions of the body (e.g., respiratory, muscular symptoms). The interplay between these two general factors moderates the affective responses to physical activity. [9].

To test the assumptions of the DMT, most studies have been conducted in the laboratory. Participants realize different intensities of bicycle or running activities using an ergometer. But, there is a lack of research on the association between momentary affects and physical activities in daily living [10]. If the association between low-intensity physical activity and affective states holds true for activities in daily living, this finding will be important for recommendations to improve older adults’ subjective well-being. Older individuals are probably more motivated to enhance the volume of activities in daily living than to follow a structured exercise or sports program [4, 11, 12]. This is important, as it has been shown in Gerontology that daily activity is a critical determinant of successful ageing.

### Affective states

Affective states and emotions represent the affective component. Together with the cognitive component (life satisfaction), they constitute the construct of subjective well-being [13]. The psychologists Osgood, Suci and Tannenbaum (1957) established affect as a multidimensional construct. In their research using semantic differentials, three dimensions were found: valence, potency and arousal. Subsequently, two dimensions were found for valence (pleasure – displeasure) and for arousal (tired – awake) [14, 15]. Schimmack and Grob [16] stated that arousal is not per se a pleasant or unpleasant feeling. Based on findings from structured equation analyses, Schimmack and Reisenzein [17] demonstrated that arousal should be differentiated into two dimensions: energy (full of energy – without energy) and excitation (calm – agitated). Thus, together with valence (pleasure – displeasure), affective states involve three affective dimensions. Besides, European researchers often favor a three-dimensional model, whereas researchers from North America often prefer a two-dimensional model [16].

With respect to attaining a positive mood, individuals benefit if they experience positive affective states more often [18]. The frequency of positive affective states is more important than their intensity. Being physically active during everyday life represents an opportunity to provoke positive affective states as often as possible. This assumption is a common hypothesis in gerontology [19, 20].

Despite the plausibility of this assumption, little research is available on how the variability of momentary affective states of older adults can be attributed to physical activities in daily living. A recently published position paper listed 22 studies that focus on the within-subject relationship between physical activity in everyday life and momentary affective states [21]. Overall, the authors cited primarily positive associations.

However, only three studies in this review address older individuals [22–24]. For example, Powell et al. [24] investigated 25 older adults (mean age = 71.4 years) who underwent joint replacement surgery within the past year. The results yielded a contradictory phenomenon: When activity values were higher, negative feelings were also higher. The negative feelings may be influenced by the joint replacement surgery provoking pain or anxious feelings. Dunton et al. [23] observed 23 healthy community-dwelling older adults (aged 50 and 76 years old) four times a day during a fortnight. The association between self-reported physical activity and positive affective states was positive. In sum, studies examining the associations between older adults’ physical activities in daily living and affects are rare. Moreover, those studies reveal contradictory findings. In addition, most studies are done in the setting of a laboratory using a standardized protocol.

The study reported here looks to real life settings, as most of older adults’ physical activity is done in daily living. According to DMT, activities in daily living are suited for the enhancement of positive affective states. Last but not least, ambulatory assessment studies should more easily generalize to everyday life, compared to controlled lab studies.

To clarify the facts an innovative ambulatory assessment design is used. Most of the few previous studies conducted a time-based ambulatory assessment. For instance, participants were randomly requested to make hourly diary entries. Fact is, that the volume of physical activity is reduced in older age. Thus, a methodological approach using a time scheduled or time triggering approach is disadvantageous as physically active episodes are rare and not linked to a time schedule. The study reported here used an ambulatory assessment with activity-triggered e-diaries. E-diary assessments commenced when the average volume of the last 10 min of physical activity exceeded a default threshold. The algorithm is published elsewhere [25]. To our knowledge, activity-triggered e-diaries have not been previously used to investigate the variability of momentary affective states attributed to physical activities in daily living.

In this study, we used the 3-dimensional model to assess affective states. As explained before, the 3-dimensional model differentiate valence, energetic arousal, and calmness, where energetic arousal stands for the physiological part (e.g. feeling tired vs. feeling awake) and calmness for the psychological part (e.g. feeling relaxed vs. feeling agitated) of the arousal dimension. We expect that the more active older individuals are, the better they will feel (valence) 10 min later. They will feel more activated and more energetic (energetic arousal). As former ambulatory assessment studies haven shown [26, 27] they will feel less calm and more agitated (calmness). The authors speculated that the relaxing effect – as could be expected focusing on laboratory or field studies with single bouts and pre-post measurements – might appear only after a time delay. It could be caused by a so called ‘rebound effect’. Subjects are exhausted and lucky having stopped their strenuous activity.

Our analyses included cross-level interactions, such as body weight, gender [28], and time of day [29]. In addition, we statistically controlled for the cognitive dimension of well-being (i.e., life satisfaction) as the affective dimension is associated with the cognitive one.

## Methods

### Selection of study participants

A randomized sample of 74 older adults adults participated in this ambulatory assessment study. The registration office of a city in the southern part of Germany provided the authors with 600 randomly chosen addresses of individuals older than 50 years. The sample was stratified by age (50 to 70 years old) and by sex (with a balanced distribution of men and women). Older people were eligible for the study if they were able to manage their daily life without restrictions by chronic discomfort (e.g. severe chronic back pain or arthritis). All of the subjects took part in the study voluntarily. They received 15 € as compensation for their participation. Following the process of institutional ethical approval, all of the participants provided written informed consent. APA ethical standards were followed in the conduct of the study.

Included in the analyses are the data of 74 older individuals. Table 1 shows main characteristics of the participants. Due to technical problems of one of the accelerometers, one complete dataset was deleted. In addition, the datasets of four more individuals were deleted because they did not provide any input into the electronic diary. When asked about the missing data by phone, these four individuals cited problems with electronic entry or their inability to hear the acoustic signal.

**Table 1:** Characteristics of study participants

	% or mean (SD)
Gender (% men)	51 %
Age	60.1 (7.1)
Marital status (% married or defacto)	65 %
Educational attainment	
● University degree	46.4 %
Work status	
● Currently working	27.5 %
● Retired	53.7 %
● Homemaker	18.7 %
Averaged activity during measurement time (milli-g/min)	73.3 (35.3)
BMI	24.7 (2.9)

### Ambulatory assessment using activity triggered e-diaries

Ambulatory assessment studies, by definition, observe participants’ natural behavior rather than manipulating their behavior. Although this feature is advantageous for understanding real aspects of everyday behavior, it may cause problems, for instance, when the variability or frequency of the target behavior (e.g., physical activity) is low. As individuals grow older, the volume of their physical activity decreases [4, 28]. To reveal meaningful associations between physical activity and its affective states, observations of low and highly active episodes should be acquired. In contrast, if a fixed or random sampling design (e.g., hourly measurement) is used in an electronic diary study, the few active episodes of older individuals will most likely be missed.

To solve this methodological problem, the present study used an innovative method, which we call an activity-triggered e-diary design (for more details see [25]). Momentary affective states were assessed via e-diaries when the accelerometer measured a volume of physical activity that surpassed a predefined activity threshold (activity > 220 milli-g; 10-min moving average) or fell below a predefined inactivity threshold (activity < 10 milli-g; 10-min moving average). In those cases, the accelerometer produces an acoustic signal.

The subjects were asked to fill out the e-diary on a commercially obtainable smartphone (an HTC Touch2) ever they heard the sound. The e-diary was programmed with MyExperience (Movisens GmbH, Karlsruhe, Germany) software. To obtain assessments of these psychological data for each individual during the most and least active episodes independent of their overall individual volume of physical activity, the thresholds were programmed to be adaptive. Therefore, if a person was very inactive, both thresholds decreased, whereas if a person was very active, both thresholds increased. To gather a sufficient quantity of data, the time interval between two e-diary assessments was limited to at least 40 min and at most 100 min. The target ratio for activity-to-inactivity episodes was established as 1:1 (see for more details [25]).

### Procedure

The study was conducted over the course of three consecutive days, beginning on Thursday and ending on Saturday. Research assistants visited the participants in their homes and explained the details of the study design. They informed the participants that they could leave the study at any time without providing a reason (written informed consent). If subjects agreed to join the study, the research assistants explained and demonstrated the usage of the accelerometer and the smart phone. The accelerometer was placed on a belt on the individual’s hip. Finally at this visit, the subjects gave written answers to questions on their demographics and how they rate their quality of life.

### Measurements

#### Actual physical activity in daily living (aPA)

We used the accelerometer varioport-e (Becker, Meditech, Karlsruhe, Germany) because it enables real-time analyses. The varioport-e measures and it stores subject’s acceleration in three axes with a frequency of 64 Hz. The raw acceleration data (milli-g) were separated offline into AC- and DC-components by using a FIR-digital filter, which a cut-off frequency of 0.5 Hz. The raw signals, DC-values and rectified AC-values were then averaged across data points for each minute during the 3-day measurement period. All offline analyses and artifact-checks were performed by using the interactive-software-package (Freiburg Monitoring System) according to a published procedure [30]. To analyze the association between aPA and affects, the aPA values during the 10 min preceding each entry into the e-diary were averaged. Multiple papers demand comparability for sensor output across manufacturers and suggest using raw acceleration (m/s^2^) [31–33]. Technically, acceleration is a physical unit expressed in m/s^2^ or as the gravitational constant g (9.81 m/s^2^). In our paper we used milli-g, which is g*1000.

#### Affective states

Momentary affective states were measured by using the Short Mood Scale, which is based on the Multidimensional Mood Questionnaire (MDMQ; [34]). MDMQ is a valid, reliable tool that can distinguish between three affect dimensions. The short scale is the only instrument that has been explicitly developed and evaluated for use in ambulatory assessment [35]. The scale contains six items. With two items each subjects assess the intensity of the three dimensions of affective states ordered as semantic differentials: *valence* (unwell vs. well, discontent vs. content), *calmness* (relaxed vs. tense, calm vs. agitated), and *energetic arousal* (tired vs. awake, without energy vs. full of energy). Subjects answered to the prompt: “At this moment, I feel…” by moving a slider from the left end (e.g., unwell) in direction of the right end (e.g., well) of the bipolar scale. Scores for each subscale were obtained by averaging the item scores, which resulted in a range from zero to five. It is worth noting that the seven-point Likert scale of the original version was changed to a six-point scale, which did not contain a mean value. This was made to force the subjects to decide on a direction for each affect dimension (e.g., unwell vs. well). Wilhelm and Schoebi [35] investigated homogeneity at the between-person and the within-subject level. The reliability coefficients for the between-person level reached 0.92 for valence and 0.90 for energetic arousal and calmness. The reliability coefficients for the within-subject level reached 0.70 for valence and calmness and 0.77 for energetic arousal. Based on these findings, the reliabilities both resulted in satisfactory internal consistencies.

#### Covariates

The moderating effects of gender, body mass index (BMI; kg/m^2^; calculated using self-reported body weight and height), and diurnal variations were assessed. To assess whether time of the day has an impact on affect dimensions, daytime was centered (time - 12). The results of the statistical analyses were controlled for linear and squared effects of time. Furthermore, the impact of general satisfaction with life, which is the cognitive component of subjective well-being, was analyzed using the Satisfaction With Life Scale (SWLS), developed by Diener, Emmons, Larsen, and Griffin [36]. Pavot and Diener [37] confirmed moderate to high internal consistencies (Cronbach’s alpha: 0.79 to 0.89). The scale contains five items (e.g., “If I could live my life over, I would change almost nothing”) each with a five-point response scale. The ratings of the five items were averaged (range: 5 to 25) to form satisfaction with one’s life.

### Data analyses

To analyze the variability of momentary affective states attributed to aPA, it is necessary to account for the data’s hierarchical structure. The multiple measures of momentary affects and aPA define the lower level of the hierarchy. These components were nested within the subjects who define the higher level of the hierarchy. Multilevel analyses were applied using the HLM 6.0 statistical program [38]. Restricted maximum likelihood estimations were used. The alpha-level was set at *p* < 0.05.

A step up approach was used [39]. First, for each affect subscale (valence, energetic arousal, calmness) in the absence of predictor variables a model was tested to separate the variance into within- and between-subject sources. An estimate of the intra-class correlations was used for it. In the second step, level-1 predictor variables (i.e., time-linear, time-squared, and aPA effects) were consecutively entered into each model of the affect subscale separately. As there were no differences between the results when time-linear and time-squared effects were included in the three models, the following analyses only controlled for time-squared effects. The final step analyzed whether valence, energetic arousal, and calmness (intercept) or the last 10 min of aPA before each e-diary entry (slope) significantly varied as a function of sex (SEX), body mass index (BMI), and general satisfaction with life (SWLS). The equations for the hierarchical levels are as follows:

1 $$ \mathrm{Level}-1:{Y}_{ti}={b}_{0i}+{b}_{1i}{\left(\mathrm{a}\mathrm{P}\mathrm{A}\right)}_{ti}+{b}_{2i}{\left(\mathsf{TIME}\hbox{-} \mathsf{SQUARED}\right)}_{ti}+{r}_{ti} $$

2 $$ \mathrm{Level}-2:{b}_{\mathit{\mathsf{0}}\mathit{\mathsf{i}}}={\gamma}_{00}\left(\mathrm{SEX}\right)+{\gamma}_{01}\left(\mathrm{B}\mathrm{M}\mathrm{I}\right)+{\gamma}_{02}\left(\mathrm{SWLS}\right)+{\mu}_{0i} $$

3 $$ \mathrm{Level}-2:{b}_{\mathit{\mathsf{1}}\mathit{\mathsf{i}}}={\gamma}_{10}\left(\mathrm{SEX}\right)+{\gamma}_{11}\left(\mathrm{B}\mathrm{M}\mathrm{I}\right)+{\gamma}_{12}\left(\mathrm{SWLS}\right)+{\mu}_{\mathit{\mathsf{1}}\mathit{\mathsf{i}}} $$

4 $$ \mathrm{Level}-2:{\mathit{\mathsf{b}}}_{\mathit{\mathsf{2}}\mathit{\mathsf{i}}}={\gamma}_{20}+{\mu}_{\mathit{\mathsf{2}}\mathit{\mathsf{i}}} $$

Level 1 calculates the within-subject effects. Equation 1 represents the subject’s response (subscript _i_) for one of the three subscales (Y_*ti*_) for any given diary entry (subscript _t_). Y_*ti*_ is defined as the average intercept of one subscale across all subjects (*b*_*0i*_) and two of the level 1 predictors: aPA (*b*_*1i*_*aPA*_*ti*_) and time-squared (*b*_*2i*_*time-squared*_*ti*_) effects. These predictors are mean-centered by groups, where the group refers to a person. By this, we disaggregate the between- and the within-subject effect [40]. The intercepts and slopes vary randomly. The random effect for the level 1 model is given by *r*_*ti*_, which is assumed to have a normal distribution, “0” mean, and a variance of σ^2^.

Level 2 expresses the between-subject effects. This level includes the fixed effects, γ of the average intercepts and slopes across all subjects: the three covariates gender (SEX), body mass index (BMI), and satisfaction with life (SWLS) and the random effects *μ*_0i,_*μ*_1i,_*μ*_*2i*_ and *μ*_*3i*_. The random effects are assumed to be multivariate and normally distributed, with both having expected values of “0”.

To clarify the magnitude of the effects, aPA and time-squared effects on the three subscales of the affective states were standardized (see Equation 5). The standard deviation was obtained from the mean of the sample of the last 10 min of aPA before each diary entry or of the time-squared effects and from the sample mean of the affect subscale. Therefore, the standardized effect was calculated as:

5 $$ \mathsf{S}\mathsf{t}\mathsf{a}\mathrm{n}\mathsf{d}\mathsf{a}\mathsf{r}\mathsf{d}\mathsf{i}\mathsf{z}\mathrm{e}\mathsf{d}\ \mathrm{e}\mathsf{f}\mathsf{f}\mathrm{e}\mathsf{c}\mathsf{t} = {b}_{\mathsf{1i}}*SD\left(\mathsf{a}\mathsf{P}\mathsf{A}\ \mathsf{o}\mathsf{r}\ \mathsf{t}\mathsf{i}\mathsf{m}\mathrm{e}\hbox{-} \mathsf{squar}\mathrm{e}\mathsf{d}\right)/\mathrm{S}\mathrm{D}\left(\mathsf{corr}\mathrm{e}\mathsf{s}\mathsf{p}\mathsf{o}\mathrm{n}\mathsf{d}\mathrm{e}\mathrm{n}\mathsf{t}\ \mathsf{a}\mathsf{f}\mathsf{f}\mathrm{e}\mathsf{c}\mathsf{t}\ \mathsf{s}\mathsf{ubscal}\mathrm{e}\right) $$

## Results

### Descriptive statistics

The 69 participants provided 1331 data points, with an average of 19.3 e-diary entries (range: 5 to 40) per subject. The average reported valence in the sample was 4.01 (SD = 0.95). The average energetic arousal was 3.55 (SD = 1.17) and the average calmness was 3.63 (SD = 1.09). The results of the intra-class coefficients were ρ_I_ = 0.40, ρ_I_ = 0.22, and ρ_I_ = 0.29 for valence, energetic arousal, and calmness, respectively. These results indicate that 60 %, 78 %, and 71 % of the respective affect subscale variance was caused by intra-individual variation (e.g., owing to situational effects).

Across the participants, the average level of aPA for the 10-min period prior to the measurement of momentary affects was 105.5 milli-g/min (SD = 137.3), ranging from 0.01 to 1307.5 milli-g. For comparison purposes, jogging episodes produce approximately 1000 milli-g/min, walking episodes produce approximately 350 milli-g/min, and sheer sitting episodes produce approximately 10 milli-g/min [21].

The average SWLS score was 3.89 (SD = 0.61), which indicated middle to high satisfaction with life. The distribution of aPA effects, time-squared effects, SWLS, and the three affect subscales allow for multilevel analyses. The fixed and random effects for each dimension of the affective states are displayed in Table 2.

**Table 2:** Fixed and random effects and variance components for valence (model 1). energetic arousal (model 2), and calmness (model 3) on actual physical activity of every day life (aPA)

Outcome	Fixed					Random						
Predictor	Coefficient	SE	t-Value	df	*p*-Value	SD	χ2	df	*p*-Value	95%-Predictive interval^a^		Slopes > 0^b^
Model 1: Valence												
Intercept	3.99	0.06	66.06	65	<0.001	1.64E-03	116.74	64	<0.001			
sex	−0.15	0.13	−0.11	65	0.91							
BMI	0.03	0.02	2.39	65	0.02							
SWLS	0.37	0.11	3.34	65	<0.001							
aPA	6.20E-05	3.37E-04	0.18	65	0.86	1.64E-03	116.74	64	<0.001	−3.15E-03;	3.28E-03	51%
aPA*sex	−6.27E-04	6.50E-04	−0.97	65	0.34							
aPA*BMI	−3.04E-04	1.22E-04	−2.48	65	0.02							
aPA*SWLS	−4.73E-04	5.03E-04	−0.93	65	0.35							
time-square	−4.38E-04	1.35E-03	−0.33	65	0.75	6.58E-03	100.77	64	0.01			
Model 2: Energetic arousal												
Intercept	3.39	0.08	40.99	65	<0.001	0.61	262.09	64	<0.001			
sex	−2.70E-01	1.70E-01	−1.59	65	0.12							
BMI	2.00E-02	3.00E-02	0.61	65	0.54							
SWLS	3.70E-01	1.40E-01	2.53	65	0.01							
aPA	1.72E-03	4.55E-04	3.79	65	<0.001	2.42E-03	121.05	64	<0.001	−3.00E-03;	6.46E-03	76%
aPA*sex	1.83E-03	8.63E-04	2.12	65	0.04							
aPA*BMI	1.20E-04	2.20E-04	0.54	65	0.59							
aPA*SWLS	−5.09E-04	6.56E-04	−0.77	65	0.44							
time-square	−9.24E-03	1.62E-03	−5.71	65	<0.001	7.34E-03	87.71	67	0.045	−2.40E-02;	5.14E-05	10%
Model 3: Calmness												
Intercept	3.78	0.08	50.29	65	<0.001	0.54	238.01	65	<0.001			
sex	4.00E-02	1.80E-01	0.24	65	0.81							
BMI	6.00E-02	2.00E-02	1.72	65	0.01							
SWLS	3.60E-01	1.20E-01	2.99	65	0.01							
aPA	−1.74E-03	4.97E-04	−3.51	65	0.01	3.06E-03	210.94	65	<0.001	−7.64E-03;	4.25E-05	28%
aPA*sex	−1.44E-03	1.13E-03	−1.27	65	0.21							
aPA*BMI	−2.92E-04	1.88E-04	−1.56	65	0.12							
aPA*SWLS	4.37E-04	8.33E-04	0.52	65	0.6							
time-square	4.20E-03	1.36E-03	3.09	1322	0.01							

^a^Based on the assumption of normally distributed regression coefficients, the 95 % predictive interval indicates the range of values between which 95 % of the regression coefficients are estimated to lie (Hox, 2010). The intervals were calculated based on a model without Level 2 predictors

^b^Based on the assumption of normally distributed regression coefficients, this value indicates the percentages of regression coefficients that are positive (Hox, 2010). The percentages were calculated based on a model without Level 2 predictors

### Affect subscale of valence

Valence was not significantly predicted by the last 10 min of aPA before each diary entry. However, 51 % of the slopes for aPA were positive, and 49 % were negative, which indicated that subjects differed with respect to their relationship between aPA and valence. To describe the reasons of the subjects’ differences, we conducted a moderator analysis.

BMI was significantly related to the within-subject effect of aPA. Older individuals with lower BMI scores were more likely to feel well and content when they were physically active (standardized effect: −0.0009). With regard to incremental contributions to variance, the effect sizes of the product-term interactions tend to be small [41].

SWLS was significantly and positively related to the intercept of valence. According to the standardized effects, if SWLS increased by 1 SD, valence would increase by 0.23 SD. This means, when older individuals felt more satisfied with their lives in general, they were more likely to feel well and content across all situations. No significant cross-level interaction for the SWLS with the effect of aPA was noted. There were no statistically significant associations between time-squared effects and gender.

### Affect subscale of energetic arousal

Energetic arousal was significantly and positively affected by the last 10 min of aPA. If subjects were more physically active during the preceding 10 min, they were more likely to feel awake and full of energy. In addition, 76 % of the slopes for aPA were positive. According to the standardized effects, if aPA increased by 1 SD, energetic arousal increases by 0.2 SD. In addition to the main effect, gender exhibited a significant cross-level interaction for the effect of aPA on energetic arousal (standardized effect: 0.00078). Women felt more energetic and awake than men did after being physical active.

The level of energetic arousal did not differ between men and women, but it was affected by SWLS. If older individuals were more satisfied with their lives in general, they were more likely to feel full of energy (standardized effect: 0.19 SD). Time-squared effects had a significant and negative effect on energetic arousal (standardized effect: 0.16 SD). Our participants were more likely to feel tired and without energy later in the day. BMI was not associated in a significant way with energetic arousal.

### Affect subscale of calmness

Calmness was significantly and negatively predicted by the last 10 min of aPA (standardized effect = −0.22). Our subjects felt agitated and tense when they were more physically active during the 10 min before each diary entry. In addition, 72 % of the slopes for aPA were negative.

SWLS was significantly and positively related to the level of calmness. According to the standardized effects, if SWLS increased by 1 SD, calmness increased by 0.2 SD. No significant cross-level interactions were observed between the SWLS and the within-person effect of aPA on calmness. Time-squared effects had a significant and positive effect on calmness (standardized effect = 0.08). Our participants were more likely to feel relaxed and calm later in the day. Gender did not exhibit any significant relationships.

## Discussion

To our knowledge, this study is the first one using an ambulatory assessment with activity-triggered e-diaries to investigate the variation of momentary affective states of older individuals that are attributed to physical activities in daily living. For most of the affective dimensions, physical activities in everyday life enhanced momentary affective states. If older individuals were more physically active in their daily lives, the more likely they felt energized (energetic arousal) and less likely they felt calm (calmness [−]). Regarding valence, the findings did not indicate a main effect of aPA. However, a significant cross-level interaction with BMI was noted here. Older individuals with lower BMI scores were more likely to feel content and well after being physically active. Additionally, older individuals with higher BMI scores were less likely to feel content and well after being active.

The findings are mainly consistent with previous meta-analyses [1–3] regarding the effect of standardized exercise programs on mental health. The known result is that physical activity provokes positive affects in older individuals. The reported activity-triggered e-diary study expanded this current evidence based on two important perspectives. First, physical activities in daily living were investigated instead of a structured exercise program. This aspect is important for health promotion and gerontology with regard to influencing mental health and increasing, or at least stabilizing, physical activity volume of older adults. Older individuals are recommended to be physically active in daily living. They will enhance affective states. Daily life activities, such as gardening, going for a stroll or completing errands by bicycle or on foot, could result in feelings of energy (energetic arousal) and excitation (calmness [−]). The experiences of energy and excitation could serve as positive reinforcements for adhering to an active life style. If an older person anticipates that positive feelings are a consequence of being physically active in everyday life, they will be motivated to be active again. Sallis and Owen [42] provided an extensive overview about different determinants to be physically active and they showed that perceived benefits of being active are positively associated with physical activity. Thus, positive outcome expectancies seems to be an important predictor in the process of altering health behaviors [43].

Second, somewhat unexpected, physical activity in everyday life did not enhance the feelings of valence. Interestingly, this association was moderated by BMI. A methodological explanation for the non-significant main effect might be plausible. The older sample in this study has relatively high ratings of valence with a relatively low variance; thus, a ceiling effect could potentially have biased the data. But, a theoretical explanation seems more likely. Older individuals exhibit lower variability in affects in general. The socio-emotional selectivity theory [44] notes that older individuals are motivated to selectively shape their life context in a way that maximizes positive experiences and minimizes negative experiences. Carstensens et al. [45] provide evidence that older individuals are emotionally more stable during their day-to-day life. An ambulatory assessment study [46] also reported low variability rates of positive affect for older individuals. These authors compared the day-to-day variability of positive and negative affect between 18 younger (20 to 30 years old) and 19 older individuals (70 to 80 years old). They reported significant age-group differences. First, older individuals reported an increased level of positive affect compared with young adults. Second, compared with young adults, older adults exhibited lower intra-individual variability in their positive and negative affects. The authors assumed that the regulation of affective states is more effective in older than in younger adults.

Despite this theoretical assumption, previous ambulatory assessment studies of older adults demonstrated that affective states could be attributed to physical activity in everyday life [12, 24, 47]. When older individuals were more physically active, they felt better and more energetic. In contrast to the current study, most of the former studies subsumed feelings of energy and happiness as positive affective states. To increase our understanding of the effects of physical activity in everyday life on momentary affects, it seems to be essential for future studies to analyze the three affect-dimensions separately.

Distinguishing valence, energetic arousal, and calmness may also be crucial for interventions that focus on improving mental health of older individuals. Based on the results of the present study, older individuals may feel energized and agitated but not better after having been physically active in everyday life. The moderator analyses indicated a significant and positive effect of BMI. As a review previously demonstrated [48], an increasing number of individuals in the population are overweight and obese because the population is growing older. To enhance the volume of daily physical activities of these individuals, it is crucial to address the potential negative impact on valence. Older individuals must be aware of strategies that can help them overcome negative affects. Otherwise, it may be that they will most likely remain inactive and sedentary and by this jeopardize their health.

In the present study, energetic arousal and calmness are both affected by the time of day, which indicates the presence of diurnal variations for these two dimensions of affective states. This finding is consistent with that of other studies that use younger [49] and middle-aged individuals [50]. Stone et al. [50] assessed different affective states every 15 min from 7 am to 9 pm. Based on their results, the scores for anxiousness, happiness, feeling rushed, and tiredness were significantly predicted by the time of day, which confirms the results from this study regarding energetic arousal and calmness.

Life satisfaction affects all of the three subscales of affective states. These effects are consistent with the theoretical assumption that both cognitive and affective dimensions of subjective well-being are interrelated [13]. In this study no significant cross-level interaction was noted. Thus at least in this sample, the relationship of physical activities in daily living with momentary affects is independent of the satisfaction of older individuals with their lives.

In addition to other facts, an important feature of this study is the activity-triggered e-diary design. As previously demonstrated [25], assessing momentary affects as a function of the volume of physical activity in everyday life increases the number of assessments during physically active episodes compared with the results of fixed or random sampling designs. Activity-triggered e-diaries maximize the variation of affective states that are based on the activities of daily living and therefore optimize the design to detect associations between both parameters [25].

Despite the powerfulness, this study has also two main limitations. First, the sample included older individuals with a relatively high level of educational achievement (i.e., high school and university degrees). Despite the randomization process, we did not achieve a normal distribution for the educational attainment of older individuals in the study that was comparable with the general population of older individuals in Germany [51]. Individuals with high educational attainments are often healthier [52], more satisfied with life [53], and more physically active [54]. The relative low BMI of the subjects endorses the possibility of a selection bias. Second, the strength of an ambulatory assessment design is the investigation of variables in real life situations. That – on the other hand – implies that it is not possible to control for potential confounders such as weather conditions, environment or interpersonal contacts. Those may have influenced the association between physical activity and affective states. Therefore, the design did not allow strong causal statements. However, the study controlled for time dependency of the independent (physical activities of daily living) and the dependent (affective states) variables by averaging the 10 min of physical activity before the affect measurement.

## Conclusion

To our knowledge, this study is the first to use an activity-triggered ambulatory assessment to analyze the variability of momentary affects of older individuals that are attributable to physical activities in daily living. Based on the findings here, physical activities in daily living are suitable to improve affective states of older individuals. In addition, the results indicated distinct effects for three subscales of affective states. Older individuals felt energized and agitated but not better after being physically active in everyday life. Thus, it seems to be essential for future studies to analyze the three affect-dimensions separately. On the basis of the socio-emotional selectivity theory, we guess that the non-significant effect on the affect dimension valence might be due to the phenomena that older individuals are emotionally more stable during their day-to-day life.

Interestingly, body mass index (BMI) and valence were associated in a significant cross-level interaction, which demonstrates BMI as a moderating variable. To enhance positive affects of older adults, these findings are substantial for future interventions. [25]
